# Diet in the management of non-dialysis dependent chronic kidney disease: perceptions and practices of health professionals

**DOI:** 10.1186/s12882-022-02790-y

**Published:** 2022-04-22

**Authors:** Stephanie Notaras, Kelly Lambert, Janette Perz, Angela Makris

**Affiliations:** 1 School of Medicine, Western Sydney University, Building 30, Campbelltown NSW, Campbelltown, 2560 Australia; 2 School of Medicine, Faculty of Science, Medicine and Health, University of Wollongong, Building 41, Wollongong, NSW 2522 Australia; 3 Translational Health Research Institute, Western Sydney University, Building 3, David Pilgrim Avenue, Campbelltown, NSW 2560 Australia

**Keywords:** Dietary interventions, Renal clinicians, Chronic kidney disease, Progression, Cross sectional survey, Dietitian, Allied health professional, Nurse, Doctor, Nephrologist

## Abstract

**Background:**

Therapeutic strategies, including dietary intervention, to target non-dialysis dependent Chronic Kidney Disease (CKD) progression have been at the forefront of recent renal research. Nephrologists and other renal health professionals are key stakeholders in the dietary management of patients with non-dialysis dependent CKD and referrals to dietetic services. The aims of this study were to explore (i) health professional perceptions regarding the role of diet in managing non-dialysis dependent CKD, and (ii) health professional practices regarding the provision of dietary advice and referrals to dietetic services.

**Methods:**

A 31-item online survey was emailed to members of professional renal networks and associations in Australia and New Zealand. Data was analysed descriptively. Categorical variables were assessed to determine associations between referral frequency, demographic variables, health professional role (non-dietetic versus dietetic) and perceptions of the role of diet.

**Results:**

Overall, 189 health professionals completed the survey. Nephrologists (42%), renal nurses (29%) and renal dietitians (24%) were the most common respondents. Non-dietetic health professionals rated the importance of diet in the management of non-dialysis dependent CKD significantly lower than renal dietitians (73% versus 98% ranked as very-extremely important, *p* = 0.002). Fifty percent of non-dietetic health professionals referred patients to renal dietetic services never or 0–25% of the time. Reasons for not referring included perceptions there is a lack of evidence that diet reduces CKD progression, perceptions that patients will not adhere to dietary recommendations, and a desire to reduce visit burden for patients. Barriers to accessing dietetic services were perceived to be significant and include lengthy wait times and inadequate dietetic staffing.

**Conclusion:**

Inconsistencies exist between non-dietetic health professionals and dietitians regarding the importance of diet in non-dialysis dependent CKD. Referral practices appear to be influenced by beliefs about the evidence base and perceptions regarding the ability of dietitians to meet referral demand. Raising awareness for non-dietetic health professionals working in nephrology regarding the evidence on diet and CKD progression is needed. An improved understanding of this evidence base may improve knowledge and referral patterns. Further, an increase in renal dietetic staffing is recommended to enhance patient access to services.

**Supplementary Information:**

The online version contains supplementary material available at 10.1186/s12882-022-02790-y.

## Introduction

Therapeutic strategies, including dietary intervention, to target Chronic Kidney Disease (CKD) progression have been at the forefront of renal research [1, 2]. In CKD Stages 1–4, dietary modification is used to manage CKD progression by targeting risk factors such as hypertension, diabetes, proteinuria and managing electrolyte imbalances and fluid overload [3, 4]. Once patients reach CKD stage 5 (end-stage kidney failure, ESKF) there are significant health care costs and a decline in patients’ physical and psychological health [5], quality of life [6], and societal productivity [1, 2].

There is clear evidence of benefit for non-pharmacological therapies such as dietetic intervention in the management of patients with CKD [7–10]. Dietetic interventions have investigated the effects of lower sodium (< 2300 mg/day) and low to moderate protein (0.6–0.8 g/kg) diets in patients with CKD Stages 3–4 [7–10]. These studies demonstrated significant reductions in systolic blood pressure control up to 11 mmHg and a 51% reduction in proteinuria. In comparison, Dapagliflozin (a SGLT2 inhibitor used to treat proteinuria) found a 26% reduction in proteinuria and a 3.5 mmHg reduction in systolic blood pressure compared to the placebo [11]. Thus dietary therapy is comparable and in some studies more effective to pharmacotherapy in managing hypertension and proteinuria with fewer side effects [7–10] but often under-utilized and under-appreciated in the management of CKD [4].

Even at later stages of non-dialysis dependent CKD (that is stages 4–5), recent research has demonstrated that access to pre-dialysis dietetic consultation was associated with a 7.5 month delay in commencing dialysis, a 37% lower risk of requiring dialysis over a four-year period, lower costs to health services and a lower number of hospital admissions [12]. Whilst these studies suggest there is substantial benefit associated with pre-dialysis dietetic consultation, the ability to access and utilize pre-dialysis services is known to vary across renal units [13]. Renal dietetic intervention prior to dialysis is not an established practice with many patients not exposed until they reach dialysis [12, 13].

CKD Stage 3 is the time where specialized nephrologist care is recommended [14] and the stage where dietetic interventions can positively impact risk progression factors [7–10]. Patients living with CKD have identified the need for dietary interventions on reducing CKD progression from earlier stages as a research priority [15]. Nephrologists and health professionals working in nephrology are key stakeholders in the dietary management of patients with non-dialysis dependent CKD and referrals to dietetic services. However, their views on the role of diet in CKD progression, their own practices regarding the provision of dietary advice, and their decision-making on the referral of patients to dietetic services are absent from the literature.

The aims of this study were to explore (i) health professional perceptions regarding the role of diet in managing non-dialysis dependent CKD from Stage 3 and (ii) health professional practices regarding the provision of dietary advice and referrals to dietetic services in Stage 3 CKD.

## Methods

### Study design, population and survey development

An online study-specific survey of renal health professionals practicing in Australia and New Zealand was conducted. The survey was developed with an experienced research team comprising of a nephrologist, two renal dietitians and a qualitative research expert. The survey questions were produced through discussions with the research team and in reference to the gaps in the literature relevant to the topic.

Data collected included demographic information, clinical experience, views on the importance of dietary management on CKD progression across all stages of the disease trajectory (Stages 1–4, pre-dialysis, dialysis, pre-transplant and post-transplant), and practice patterns for referring patients to renal dietetic services from CKD Stage 3. The survey was peer reviewed with three renal health professionals (two nephrologists and a renal nurse). Minor changes were recommended to improve the clarity and readability of the questions.

### Survey administration and data collection

A final 31-item online survey (see Additional file 1) was administered using Qualtrics software. The survey was distributed via email from the Australia New Zealand Society of Nephrology (ANZSN) to its registered members (*n* = 770) and the New South Wales Agency of Clinical Innovation (ACI) renal network members (*n* = 449). Renal health professionals were likely to be members of both ANZSN and ACI. Two emails were sent four weeks apart and the survey was open for completion from 19 January 2021 to 15 July 2021.

Participants undertook the survey anonymously and implied consent was obtained upon survey completion and submission. Ethical approval to complete this study was obtained through the South Western Sydney Local Health District Human Research Ethics Committee (HREC approval number—2020/ETH01309).

### Statistical analysis

Descriptive statistics are presented as counts and percentages. There was no imputation of missing data. Data was analysed separately for renal dietitians and non-dietetic health professionals to better understand the views of non-dietetic renal health professionals.

Categorical variables were assessed using the Fisher’s Exact test to determine the associations between referral frequency and demographics such as gender, age categories, location of training, location of practice, health professional role and years of practice. Referral frequency was split into two groups: non-dietetic health professionals that never refer or refer patients to dietitians 0–25% of the time and those that refer patients to dietitians 26–100% of the time or always.

Stepwise backward binomial logistic regression was used to determine which variables were associated with referral frequency. Demographic variables found to be significant between the two groups such as gender, location of training, location of practice, health professional role were included, along with age and years of practice. Probabilities for entry or removal of variables from the model were 0.05 and 0.1, respectively. Odds ratios with 95% confidence intervals are presented. Simple thematic analysis was used to analyse free text responses on dietary advice provided to patients and then grouped into relevant categories for interpretation. The data was analysed using Statistical Package for the Social Sciences (SPSS) (Version 28; IDM Corp, Armonk, NY). A *p*-value less than 0.05 was considered statistically significant.

## Results

### Demographic characteristics

A total of 190 participants completed the survey (25% response rate, using a denominator of 770 potential participants, as both mailing lists contained many of the same health professionals). One participant was excluded from the analysis as they were an administrative officer and not a renal health professional. A total of 189 participants were included in the analysis and demographic characteristics are shown in Table 1. Overall, 75% of participants completed the entire survey with a significantly higher proportion of males finishing the survey compared to females (90% vs 70%, *p* = 0.01). There were no differences in rate of completion according to age, years of practice and health professional type.

**Table 1:** Demographic characteristics of study participants

Characteristic	All (*n* = 189)	Renal health professionals (*n* = 144)	Renal Dietitians (*n* = 45)	*P*-Value
Age				< 0.001*
20–29 years	19 (10)	9 (6)	10 (22)	
30–39 years	54 (28)	34 (24)	20 (44)	
40–49 years	45 (24)	36 (25)	9 (20)	
50–59 years	45 (24)	42 (29)	3 (7)	
60 years and over	26 (14)	23 (16)	3 (7)	
Gender				
Female	144 (76)	101 (70)	43 (96)	< 0.001*
Role				
Nephrologist	80 (42)	80 (56)	n/a	n/a
Nephrology Trainee	6 (3)	6 (4)	n/a	n/a
Nurse	54 (29)	54 (37)	n/a	n/a
Dietitian	45 (24)	n/a	45 (100)	n/a
Other	4 (2)	4 (3)	n/a	n/a
Years of renal practice				0.003*
0–9 years	80 (42)	51 (36)	29 (65)	
10–19 years	46 (24)	35 (24)	11 (24)	
20–29 years	30 (16)	28 (19)	2 (4)	
30–39 years	26 (14)	23 (16)	3 (7)	
40 years or more	7 (4)	7 (5)	0	
Type of practice				
Public hospital	182 (96)	137 (95)	45 (100)	1
Private clinics	35 (19)	29 (20)	6 (13)	0.38
Academic	13 (7)	12 (8)	1 (2)	0.31
Clinician and researcher	34 (18)	30 (21)	4 (9)	0.78
Trainee	7 (4)	7 (5)	n/a	n/a
Location of training				0.064
Australia	169 (89)	119 (83)	32 (71)	
New Zealand	17 (9)	7 (5)	7 (16)	
Overseas	3 (2)	17 (12)	6 (13)	
Practice location				
Metropolitan	133 (70)	101 (70)	32 (71)	1
Outer metropolitan	32 (17)	25 (17)	7 (16)	1
Rural	49 (26)	37 (26)	12 (27)	1
Subspeciality				
Transplant	16 (8)	16 (11)	n/a	n/a
Dialysis	41 (21)	41 (28)	n/a	n/a
General nephrology	44 (23)	44 (31)	n/a	n/a
Chronic kidney disease	19 (10)	19 (13)	n/a	n/a
All of the above	16 (8)	16 (11)	n/a	n/a
Nutrition	45 (24)	0	45 (100)	n/a
Other	8 (4)	8 (6)	n/a	n/a
Type of patients				
Adults	182 (96)	138 (96)	44 (98)	
Children	4 (2)	4 (3)	0	n/a
Both	3 (2)	2 (1)	1 (2)	
Health professionals available in the respondent’s unit				
Nurse	179 (95)	137 (95)	42 (93)	3.63
Dietitian	179 (95)	138 (96)	45(100)	2.53
Social Worker	165 (87)	127 (88)	38 (84)	1
Pharmacist	130 (67)	104 (72)	26 (58)	0.06
Psychologist	85 (45)	64 (44)	21 (47)	0.86
Occupational Therapist	62 (33)	49 (34)	13 (29)	0.59
Other	16 (8)	9 (6)	7 (16)	0.65
Unsure	3 (2)	1 (1)	2 (4)	0.14

^*^Data is presented as count (percentage). N/a indicates not applicable. Nurse includes those with specialist qualifications (for example, nurse practitioner, clinical nurse consultant, clinical nurse specialist). *Indicates p-value < 0.05

### Perceptions on the role of diet in CKD progression

**Fig. 1:**
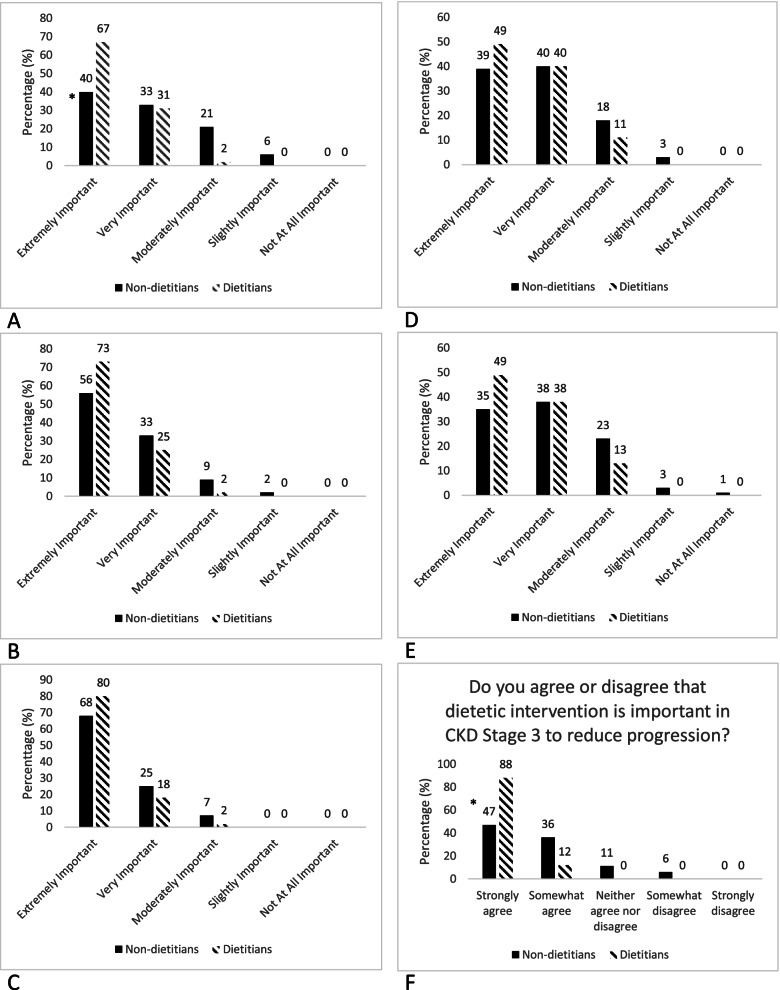
Renal health professional perceptions about the role of diet in varying stages of CKD. **A**: Comparison of perceptions about the role of diet in CKD Stages 1–4 between non-dietetic health professionals and dietitians. *P-value = 0.002. **B**: Comparison of perceptions about the role of diet in pre-dialysis between non-dietetic health professionals and dietitians. *P*-value = 0.15. **C**: Comparison of perceptions about the role of diet in dialysis between non-dietetic health professionals and dietitians. *P*-value = 0.25. **D**: Comparison of perceptions about the role of diet in pre-transplant between non-dietetic health professionals and dietitians. *P*-value = 0.38. **E**: Comparison of perceptions about the role of diet in post-transplant between non-dietetic health professionals and dietitians. *P*-value = 0.27. **F**: Comparison of perceptions about impact of dietetic intervention in CKD between non- dietetic health professionals and dietitians. *P-value < 0.001

Overall, participants perceived that diet was extremely important in the management of CKD. The relative importance of diet according to stage did not differ between pre-dialysis, dialysis, pre-transplant and post-transplant, and ranged from 38% rating diet as extremely important post-transplant to 71% in the dialysis population (Fig. 1). Perceptions of the importance of diet in CKD stages 1–4 did vary significantly according to profession, with 98% of renal dietitians (*n* = 45) ranking diet as extremely or very important in CKD Stages 1–4 compared to 73% in non-dietetic health professionals (*n* = 144, *p* = 0.002) (Fig. 1A).

The highest rankings of the role of diet in managing CKD Stages 1–4 (data not shown) were found for female non-dietetic health professionals (*p* < 0.001), those trained in Australia (*p* = 0.003) and those working in non-metropolitan regions (*p* = 0.03). No other differences in perceptions about importance of diet or stage of CKD were apparent.

Figure 1F compares perceptions on the impact of diet in CKD progression according to profession. Non-dietetic health professionals differed significantly in their perception, with 47% of non-dietetic health professionals indicating they strongly agree diet can impact progression compared to 88% of dietitians (*p* =  < 0.001).

### Practices regarding the provision of dietary advice

Survey participants reported that patients are actively engaged in asking for dietary advice. Table 2 shows that over 65% of patients ask about diet more than half the time; and that most non-dietetic health professionals indicate that the correct diet can reduce progression (67%) (Table 2). Non-dietetic health professionals used blood tests (67%), blood sugars (60%) and blood pressure (49%) to help triage the need for dietary advice. Fifty eight percent of non-dietetic health professionals gave dietary advice to all patients. The most common types of dietary advice provided to patients were about low salt diets (65%), low potassium and phosphate (34%) and fluid (20%). A lower protein diet (25%) and weight management advice (21%) were also reported. This dietary advice was provided by non-dietetic health professionals verbally (50%), followed by both verbally and written (48%). Table 2).

**Table 2:** Dietary advice provided by non-dietetic health professionals to patients with CKD Stage 3

Question	n (%)	Question	*n* (%)
How often do your patients ask about diet? (*n* = 144)		Dietary advice provided (*n* = 111)	
Never	1 (1)	Low salt diet	72 (65)
Sometimes	48 (33)	Lower protein diet	28 (25)
Half the time	35 (24)	Low potassium/phosphate diet	38 (34)
Most of the time	49 (34)	Glycaemic control	16 (14)
Always	11 (8)	Weight management	23 (21)
What do you say to patients about diet and CKD progression? (*n* = 111)		Increase plant foods	13 (12)
Diet has no impact	2 (2)	General healthy eating	18 (16)
The right diet can reduce progression	74 (67)	Avoid processed foods	9 (8)
Nothing	25 (22)	Fluid recommendations	33 (30)
Other	10 (9)	Other	33 (30)
Determinants for dietary advice (*n* = 111)		No advice given	14 (13)
Blood test results	99 (67)	How is dietary advice given? (*n* = 111)	
Adequacy of BP	71 (49)	Verbally	56 (50)
Adequacy of BSLs	86 (60)	Verbally and written	53 (48)
Individualised—based on assessment	11 (8)	Written	6 (5)
Give advice to all patients	83 (58)	Nil advice given	4 (4)
No dietary advice given	6 (4)	Refer to dietitian	65 (59)
Source of dietary information (*n* = 111)		Most challenging aspects of giving dietary advice (*n* = 109)	
Online	54 (49)	Patient motivation to change	55 (50)
Nephrology training	48 (43)	Time restraints	40 (37)
Dietetics department	90 (81)	Patient's health literacy	24 (22)
Self-learning	57 (51)	Own knowledge	8 (7)
Conferences	43 (39)	Patient's current knowledge	5 (5)
Renal nutrition guidelines	60 (54)		

*CKD* chronic kidney disease, *BP* blood pressure. *BSLs* Blood sugar levels, Some variables may have missing data and not add up to the total participant response rate. Respondents could select more than one response except for two questions: (1) how often patients ask about diet? and (2) what you say to patients about diet and CKD progression?

Data are presented as counts (percentages)

The most challenging aspects of dietary advice provision to patients that non-dietetic health professionals experienced were: patients’ motivation to change (38%), time restraints (28%) and patients’ health literacy levels (16%)(Table 2).

### Practices regarding referrals to dietitians

One in ten (11%) (Table 3) non-dietetic health professionals never referred patients with CKD Stage 3 to a renal dietitian. In contrast 29% of non-dietetic health professionals referred all or most patients. When asked who was the preferred provider for dietary advice, 88% of non-dietetic respondents reported a renal dietitian. Overall, 78% of renal health professionals had a dedicated renal dietitian in their team and did not differ between referring groups.

**Table 3:** Referral practices by non-dietetic health professionals to dietetic services for patients with CKD Stage 3

Question	*n* (%)	Question	*n* (%)
How often do you refer patients to a renal dietitian in CKD Stage 3 (*n* = 111)		Reasons for referring patients to a dietitian (*n* = 104)	
Never	12 (11)	Patients not adhering to advice	26 (25)
0–25% of the time	46 (41)	Patient request	60 (58)
26–50% of the time	21 (19)	Reduce risk of CKD progression	66 (63)
51–75% of the time	9 (8)	Treat malnutrition	45 (43)
76–99% of the time	2 (2)	Oral nutrition support	51 (49)
I refer all patients	21 (19)	Manage fluid overload	36 (35)
How often do patients initiate a referral to a renal dietitian? (*n* = 144)		Electrolyte/weight management education	11 (11)
Never	20 (14)	Factors perceived to enhance referrals to renal dietitians (*n* = 110)	
Sometimes	93 (64)	Dedicated dietitian	74 (67)
Half the time	21 (15)	Evidence of positive clinical outcomes from DI	62 (58)
Most of the time	9 (6)	Dietitian's skills and experience	56 (51)
Always	1 (1)	Hearing positive feedback from patients	55 (50)
What is your preference for who provides dietary advice? (*n* = 111)		Patient interest	64 (58)
Nephrologist	4 (4)	Shorter waiting times to see dietitian	51 (46)
Renal Dietitian	98 (88)	Suggestions for improvements to provision of renal dietetic advice (*n* = 109)	
Renal Nurse	0	Additional training (renal dietary information, counselling skills)	50 (46)
Any renal team member	9 (8)	Better written resources	70 (64)
Reasons for not referring patients (*n* = 107)		All patients to be referred to dietitian once diagnosed with CKD	61 (56)
I can give advice	13 (12)	Better service provision (frequent appointments, more dietitians)	60 (55)
Patient declined referral	66 (62)	Would you refer patients to a renal dietitian from CKD Stage 3 if the service was available? (*n* = 111)	
Visit burden for patients	35 (33)	Yes	93 (84)
Do not think patients will adhere to advice	9 (8)	No	3 (3)
Significant waiting times to see dietitian	36 (34)	Unsure	15 (14)
Poor service provision	9 (8)		
Not enough evidence that diet works in CKD	12 (11)		
Other	12 (11)		

*DI* dietetic intervention. Some variables may have missing data and not add up to the total participant response rate. Respondents could select more than one response except for two questions: (1) how often they refer patients with CKD Stage 3 to a dietitian? and (2) how often patients initiate a referral to a dietitian?

^a^Data is presented as count (percentage).

The most common reasons for referring patients with CKD Stage 3 to dietetic services were to reduce the risk of CKD progression (63%), when requested by patients (58%), to offer oral nutrition support (49%) and to treat malnutrition (43%) (Table 3). The most common reasons for not referring patients to dietetic services were patient decline (62%), significant waiting times to see a renal dietitian (34%) and the health professional’s perception that it would add to the patient’s visit burden (33%). Health professionals reported several factors that could enhance referrals to dietetic services. These included having a dedicated dietitian for patients with CKD Stage 3 (67%), evidence of positive outcomes from dietetic intervention (58%) and patient interest (58%). Health professionals suggested that better dietary related written resources (64%), improved service provision through increased dietetic staffing and more frequent appointments (55%), and the ability for all patients to be referred to dietetic services from CKD diagnosis (56%) could improve dietetic services.

When analysed based on referral frequency, the lower referring health professional group (never referred or referred 0–25% of the time) were significantly more likely to refer to a renal dietitian if their patient requested it compared to higher referring health professional group (*p* = 0.03). Health professionals in the higher referring group were significantly more likely to refer their patients to a renal dietitian to reduce the risk of CKD progression (*p* < 0.001), if they required nutrition supplements (*p* = 0.02) or to manage nutrition related symptoms (*p* = 0.01).

Health professionals in the lower referring group were significantly less likely to refer their patients to a renal dietitian due to their perception that there is not enough evidence that diet works to reduce CKD progression (*p* < 0.001), to reduce the visit burden for patients (*p* = 0.02), believing patients will not adhere to dietary advice (*p* = 0.03) and if patients declined (*p* = 0.03). The lower referring health professional group rated the importance of the role of diet in CKD Stages 1–4 significantly lower (*p*-value < 0.001) and were in a lower agreeance that dietetic intervention can help to reduce CKD progression (*p*-value < 0.001) compared to the higher referring health professional group. They also reported that evidence of positive clinical outcomes from dietetic intervention on CKD progression would significantly enhance referrals to renal dietitians compared to the higher referring group (*p* = 0.03).

Health professionals in the higher referring group rated better service provision (increased frequency of appointments and enhancements in dietetic staffing) as a significant factor that could improve how dietitians provide dietary advice to patients significantly higher than those in the lower referring group (*p* = 0.007).

The lower referring group had a significantly higher proportion of male health professionals (*p* = 0.047) and nephrologists (*p* = 0.01) compared to the higher referring group. However, once adjusted for other variables in a binomial logistic regression, the only significant predictor for referral frequency was the health professional’s role, with non-nephrologists 3.07 times more likely to refer patients to renal dietetic services compared to nephrologists (95% CI 1.36–6.92; *p* = 0.007) (Table 4). Gender (*p* = 0.40), location of training (*p* = 0.27), location of practice (*p* = 0.55) and years of practice (*p* = 0.17) were not significant independent predictors for referral to a dietitian.

**Table 4:** Factors predicting referral to dietetic services for patients with Stage 3 CKD

Variable	Adjusted Odds Ratio	95% CI	P-Value
Age of health professional	0.62	0.12–6.13	0.65
Female gender	1.52	0.57–4.05	0.40
Health professional role (nephrologist versus non-nephrologist)	3.07	1.36–6.92	0.007*
Location of training (Australia versus overseas)	0.55	0.20–1.57	0.27
Location of practice (non-metropolitan versus metropolitan)	0.75	0.30–1.89	0.55
Years of practice	0.4	0.08–4.97	0.17

^*^Indicates *p*-value < 0.05

## Discussion

This study explored the perspectives of health professionals on the role of diet in managing CKD (non-dialysis dependent), particularly in stage 3. Most patients with CKD Stage 3 ask their health professionals about the role of diet and renal health professionals believe diet can reduce CKD progression. Despite this, significant inconsistencies were found in the referral patterns between health professionals and the reasons they choose or choose not to refer patients to renal dietetic services. A perception of a lack of positive outcomes from dietetic intervention on CKD progression, poor patient adherence to dietary advice and avoiding patient visit burden were reported as the main factors for a lower referral pattern. Therefore, the perceptions of health professionals influence whether their patients are able to access and receive specialized dietetic care for CKD. Referral “gatekeeping” has been explored in primary health care for conditions such as dyslipidaemia, hypertension and obesity [16, 17]. Common reasons for lower referral rates to dietetic services were a lower awareness of the additive benefits of dietary interventions to pharmacological treatment, other medical priorities requiring attention and the perception that patients were not ready to change their eating behaviours [16, 17].

It remains unclear from the results of this study why half of the non-dietetic health professionals, in particular nephrologists, perceive there is a limited role for diet in CKD progression given the available evidence. This knowledge gap has important implications for translation into clinical practice as nephrologists are often the key stakeholders for patient referrals to dietetics in earlier stages of CKD. This needs to be explored so that therapeutic options for patients to delay CKD progression are maximized and standardized across non-dietetic health professionals. Additional awareness raising strategies for renal health professionals or changes to current renal models of care may assist with addressing this knowledge gap. For example, embedding dietetic services as standard care for patients with CKD Stage 3 might increase confidence and the profile of how dietetic care may impact progression.

The health professionals in this study raised the issue of patient adherence to dietary recommendations as a factor for not referring patients to dietetic services. Adherence is influenced by many elements including health literacy, patient understanding of the benefits of the intervention and the way the intervention is delivered [18–20]. In this study, half of the non-dietetic health professionals provided verbal dietary advice to patients without the support of written information. However, patients with CKD have identified written resources to be helpful in implementing dietary behaviour change [19, 20]. Findings from an integrative systematic review into patient adherence to renal dietary recommendations in CKD Stages 4–5 found it to be suboptimal at 31.5% [18]. However, 84% of the studies were with patients already on dialysis whose dietary needs are far more complex than patients with CKD Stages 3, including considerations of potassium, phosphate, sodium and protein. Adherence rates may be higher in patients with earlier stages of non-dialysis dependent CKD as the dietary changes required are less complicated. Patients on dialysis have reported that if they had appreciated the impact of diet on reducing CKD progression, they would have been more inclined to make the necessary dietary changes earlier in their CKD journey [21].

Visit burden is a documented issue for patients with CKD, especially those with multiple co-morbidities [22]. However, patients have reported they want earlier access to dietary interventions and dietetic services to help reduce CKD progression [23, 24]. They have also ranked treatments including diet as a top priority for renal research, highlighting that any treatment associated with reducing CKD progression as a priority not to be overlooked [15]. A qualitative study into the experiences of patients in the pre-dialysis stage of CKD (eGFR of < 20 ml/min/1.73^2^) found that avoiding or delaying dialysis was the only motivating factor for changing dietary behaviours [19]. Although delaying the need to commence dialysis is beneficial to patients, dietetic intervention during the predialysis stage is unlikely to halt CKD progression as it may if changes are advised and implemented from CKD Stage 3 [25]. Thus, patient-centered care involves providing patients with the option to accept or decline renal dietetic care, instead of the decision being made for them. Research has shown that a physician’s encouragement or ambivalence towards dietetic intervention influences whether patients continue or cease treatment with dietetic services, respectively [26]. This highlights the impact physicians have in shaping patients’ perception of the positive effect dietary changes can have on disease progression and possibly adherence to dietary changes [19, 26].

It well documented that renal dietetic staffing resources in Australia and worldwide are well below the recommendations for staff to patient ratios [18, 27]. For health professionals in the higher referral group, significant renal dietetic clinic waiting times was a reason for not referring patients to the service. Further, these health professionals reported that improving dietetic service provision through shorter waiting times, more frequent appointments and more renal dietetic staffing would significantly enhance patient referrals. Patients with CKD have also reported access to renal dietetic services and appointment frequency to be a positive factor to dietary behaviour change adherence [19], challenging the perception that dietetic services may add to the visit burden patients often experience.

Although improving renal dietetic staffing in renal units is recommended to provide optimal care to patients, this may not improve patient access if renal health professionals are not utilizing these services. Raising awareness in renal health professionals on the evidence of the role of diet in CKD management is imperative to provide consistency in patient care and uptake of renal dietetic services. Qualitative research to further explore the perspectives of renal health professionals will help to inform future strategies to improve nephrologists’ awareness of dietary education, their perception of the role of diet and patient access to renal dietetic services.

This study had limitations that may limit its generalisability. Participants were mainly recruited from Australia, therefore transferability to other countries and their health systems is uncertain. The response rate was 25% and the results may not be representative of the general renal health professional workforce. However, a response rate of 20% is considered common for health professionals based online surveys [28]. Strengths of the study included a broad representation of key renal stakeholders on the role of diet in CKD management including medical, allied health and nursing health professionals. Further, there is a lack of studies investigating the perspectives of these stakeholders and the results from this study can inform future initiatives and advocacy work to help people living with CKD access dietetic services.

## Conclusion

This is the first study to investigate the perspectives of renal health professionals on the role of diet in CKD management (non-dialysis dependent), the dietary advice they provide to patients and their referral patterns. Inconsistencies were found between the referral patterns of renal health professionals to renal dietetic services and their perceptions of the importance of the role of diet in CKD Stages 1–4. Health professional education to enhance their knowledge of the evidence in positive outcomes from dietetic intervention in CKD Stages 3–4 is suggested to improve referral patterns and patient access to renal dietitians. Further, an increase in renal dietetic staffing is recommended to facilitate interventions that can assist with reducing CKD progression.

## Supplementary Information

**Additional file 1.** Renal health professional survey. Thirty one-item online survey used tocomplete the study.

## Data Availability

The datasets generated and/or analysed during the current study are not publicly available but are available from the corresponding author on reasonable request.

## Abbreviations

CKD: Chronic Kidney Disease
ESKF: End-Stage Kidney Failure
