# Supplementary material for: Diet in the management of non-dialysis dependent chronic kidney disease: perceptions and practices of health professionals
Source: BMC Nephrol. 2022 Apr 22;23:158. doi: 10.1186/s12882-022-02790-y (PMC9034530; doi:10.1186/s12882-022-02790-y)
Supplement: Supplementary file 1 — Additional file 1. Renal health professional survey. Thirty one-item online survey used tocomplete the study. [file 12882_2022_2790_MOESM1_ESM.pdf]

Default Question Block

Investigator: Stephanie Notaras (Phd Candidate from Western Sydney University and Senior Renal Dietitian from Liverpool Hospital, NSW, Australia).

The aims of this online survey are to explore the views and experiences of clinicians treating renal disease, on the role of diet in managing CKD, in particular Stage 3. You will be asked questions about your practice patterns, providing dietary advice, your views on dietetic services and how renal dietetic services could be improved.

The survey will take 5-10 minutes to complete. Your responses are anonymous and your participation is voluntary. You may withdraw at any time throughout the survey. Once your survey has been submitted it will not be possible to cancel the submission or revoke your consent.

By completing this survey, you consent to the information you provide being analysed and reported anonymously.

Ethics approval has been obtained through the South Western Sydney Local Health District Human Research Ethics Committee (HREC approval number - 2020/ETH01309). You are welcome to discuss your participation in this study with the lead investigator (please contact stephanie.notaras@health.nsw.gov.au).

Thank you for participating in the survey. We appreciate your input!

What is your age?

- ○ 20-29 years
- ○ 30-39 years
- ○ 40-49 years
- ○ 50-59 years
- ○ 60 years or over

What is your gender?

- ○ Male
- ○ Female
- ○ ______ Other (please specify)

- ○ Prefer not to answer

What is your role?

- ○ Nephrologist
- ○ Nephrology Trainee
- ○ Nurse
- ○ Dietitian
- ○ Social Worker
- ○ Researcher
- ○ ______ Other (please specify)

How many years have you practiced in renal?

- ○ 0-9 years
- ○ 10-19 years
- ○ 20-29 years
- ○ 30-39 years
- ○ 40 years or more

Type of practice (please select all relevant)

- ☐ Public hospital
- ☐ Private clinics
- ☐ Academic
- ☐ Clinician and researcher
- ☐ Trainee

Where did you complete your nephrology training or are currently completing your training?

- ☐ Australia
- ☐ New Zealand
- ☐ ______ Other (please specify)

Where are you currently practicing?

- ○ Australia
- ○ New Zealand
- ○ ______ Other (please specify)

Type of practice location (please select all relevant)

- ☐ Metropolitan
- ☐ Outer metropolitan
- ☐ Rural
- ☐ ______ Other

Subspecialty (please select most relevant)

- ○ Transplant
- ○ Dialysis
- ○ General nephrology
- ○ Diabetes
- ○ Chronic kidney disease
- ○ Interventional
- ○ Obstetrics
- ○ All of the above
- ○ ______ Other (please specify)

What type of clients do you see in your practice?

- ○ Adults
- ○ Children
- ○ Both adults and children

Which of the following clinicians are part of your renal service? (please choose all relevant)

- ☐ Nurse
- ☐ Dietitian
- ☐ Social Worker
- ☐ Pharmacist
- ☐ Psychologist

- ☐ Occupational Therapist
- ☐ ________ Other (please specify)
- ☐ Unsure

In your opinion, how important is the role of diet in the management of CKD at various stages?

	Extremely Important	Very important	Moderately important	Slightly important	Not at all important
CKD Stages 1-4	○	○	○	○	○
Pre-dialysis	○	○	○	○	○
Dialysis	○	○	○	○	○
Pre-transplant	○	○	○	○	○
Post-transplant	○	○	○	○	○

Do you perceive it is your role to ask about diet?

- ○ Yes
- ○ No
- ○ Unsure

How do you determine which patients you give dietary advice to? (please choose all relevant)

- ☐ Blood test results - kidney function, electrolytes, HbA1C
- ☐ Adequacy of blood pressure
- ☐ Adequacy of blood sugar control
- ☐ I give dietary advice to all patients
- ☐ I do not give dietary advice to patients
- ☐ ________ Other (please specify)

How often do your patients ask you about diet?

- ○ Never
- ○ Sometimes
- ○ About half the time
- ○ Most of the time
- ○ Always

How often do patients initiate a referral to a renal dietitian?

- Never
- Sometimes
- About half the time
- Most of the time
- Always

Block 2

The following questions are about your practice with patients who have CKD Stage 3.

Please list the dietary advice you give patients with CKD Stage 3.

Do you agree or disagree that dietetic intervention is important to reduce CKD progression?

- Strongly agree
- Somewhat agree
- Neither agree nor disagree
- Somewhat disagree
- Strongly disagree

What do you say to patients about the role of diet in CKD Stage 3?

- Diet does not impact CKD progression
- The right diet can reduce CKD progression
- I do not say anything
- Other (please specify)

How do you provide the advice? (please choose all relevant)

- Verbally
- Verbally and written resources
- Written resources

- [ ] I do not give dietary advice
- [ ] I refer them to a dietitian
- [ ] ______ Other (please specify)

Where do you get your dietary information? (please select all relevant)

- [ ] Online resources
- [ ] Nephrology training
- [ ] Dietetics department/renal dietitian
- [ ] Self learning
- [ ] Conferences
- [ ] Renal nutrition guidelines
- [ ] ______ Other (please specify)

What are the 5 most challenging aspects of giving dietary advice to patients? (please rank them from 1-5, with 1 being the most challenging)

	1	2	3	4	5	6	7	8
Own knowledge	○	○	○	○	○	○	○	○
Own counselling skills	○	○	○	○	○	○	○	○
Patient's motivation to change	○	○	○	○	○	○	○	○
Patient's current knowledge	○	○	○	○	○	○	○	○
Patient health literacy	○	○	○	○	○	○	○	○
Time restraints	○	○	○	○	○	○	○	○
Difficult diet to explain	○	○	○	○	○	○	○	○
Other (please specify) ______	○	○	○	○	○	○	○	○

Who would you prefer to give your patients dietary advice?(please rank from 1-4, with 1 being the most preferred)

	1	2	3	4	5
Nephrologist	○	○	○	○	○
Renal dietitian	○	○	○	○	○
Renal nurse	○	○	○	○	○
Any member of the renal team	○	○	○	○	○
Other (please specify) ______	○	○	○	○	○

If you have a dietitian in your team, please tell us about their role.

- General dietitian - no sub-specialist qualification
- Renal specialist dietitian
- Unsure
- Do not have a dietitian in the team

How often to you refer your patients to see a renal dietitian when they have CKD Stage 3?

- Never
- 0-25% of the time
- 26-50% of the time
- 51-75% of the time
- 76-99% of the time
- I offer a referral to all my patients

What are the reasons for referring patients with CKD Stage 3 to a renal dietitian? (please select all relevant)

- Patients not adhering to the advice I have given
- Patient request
- To reduce the risk of CKD progressions through dietary changes
- To treat malnutrition
- To advice for oral nutrition support (for example, supplements)
- To manage fluid overload
- To manage blood sugars
- To manage nutrition related symptoms (for example, reduced appetite, taste changes, nausea)
- ____________ Other (please specify)

What are the reasons for not referring patients to a renal dietitian? (please select all relevant)

- I can give dietary advice
- Patient declined referral
- Visit burden for patients
- Do not think patient will adhere to dietary advice
- Significant waiting times to see a dietitian

- [ ] Not satisfied with current or past dietitians
- [ ] Negative patient feedback about renal dietitian
- [ ] Not enough evidence that diet works in CKD
- [ ] Do not believe it will affect outcomes
- [ ] ______ Other (please specify)

What would enhance referrals to renal dietetics? (please select all relevant)

- [ ] Dedicated dietitian
- [ ] Evidence of positive clinical outcomes from dietetic intervention
- [ ] Dietitian's skills and experience
- [ ] Hearing positive feedback from patients
- [ ] Patient interest
- [ ] Shorter waiting times
- [ ] ______ Other (please specify)

What do you think could improve how dietitians provide dietary advice to patients? (please select all relevant)

- [ ] Training for dietitians (dietary information, counselling skills)
- [ ] Better resources (for example, meal plans, lists of what to eat and what to avoid)
- [ ] All patients to be referred to a dietitian once diagnosed with CKD
- [ ] Frequent appointments with dietitian
- [ ] ______ Other (please specify)
- [ ] No improvements needed

If your unit had a dedicated CKD Stage 3 renal dietetics clinic, would you refer your patients to this service?

- ○ Yes
- ○ No
- ○ Unsure

Would you be interested in participating in a short phone or video interview about your views and experiences on the role of diet in CKD and renal dietetic services?

The purpose of the interview is to help us design health services that would better meet clinicians' and patients' needs.

- Yes
- No

Once you press next, you will submit the survey. A link will appear for you to click if you would like to provide your details to be contacted for an interview. Your responses to the survey will remain anonymous.

Powered by Qualtrics
